# Effects of minimally invasive procedures for evacuation of intracerebral hematoma in early stages on MMP-9 and BBB permeability in rabbits

**DOI:** 10.1186/1471-2377-14-85

**Published:** 2014-04-17

**Authors:** Guofeng Wu, Jing Shi, Fan Wang, Likun Wang, Anrong Feng, Siying Ren

**Affiliations:** 1 Emergency Department of Affiliated Hospital, Guiyang Medical College, No. 28, Guiyijie Road, Liuguangmen, Guiyang City, Guizhou Province, 550004 PR China; 2 Department of Neurology of Affiliated Hospital, Guiyang Medical College, No. 28, Guiyi Road, Liuguangmen, Guiyang City, Guizhou Province, 550004 PR China

**Keywords:** Cerebral hemorrhage, Minimally invasive procedures, MMP-9, Blood brain barrier, Neurological deficit score

## Abstract

**Background:**

The effects of performing a minimally invasive procedure at different stages after intracerebral hemorrhage on perihematomal MMP-9 expression and blood–brain barrier (BBB) permeability were evaluated.

**Methods:**

Sixty rabbits were randomly distributed into a model control group (MC group, 30 rabbits) or a minimally invasive group (MI group, 30 rabbits). A model of intracerebral hemorrhage was established in the MC and MI group. In the MI group, the intracerebral hematoma was evacuated by stereotactic minimally invasive procedures over 6 hours (6 rabbits), 12 hours (6 rabbits), 18 hours (6 rabbits) 24 hours or 48 hours (6 rabbits) following successful induction of intracerebral hemorrhage. The same procedure was performed in the MC group at the same time point but without evacuating the hematoma. All the animals were sacrificed within two weeks after the hematoma was surgically evacuated. A neurological deficit score was determined, and the perihematomal MMP-9 level and the BBB permeability were measured.

**Results:**

The neurological deficit score, perihematomal MMP-9 level and BBB permeability of the MI group decreased significantly compared to the MC group. Performing the MI procedure 6–12 h after intracerebral hemorrhage showed the most favorable outcome.

**Conclusions:**

Regarding the pathophysiological changes surrounding the hematoma, the optimal time window of performing MI procedures for the intracerebral hematoma evacuation might be within 6–12 h after hemorrhage.

## Background

Spontaneous intracerebral hemorrhage (ICH) remains the least treatable form of stroke. It is associated with high rates of morbidity and mortality. Evidence-based medical therapies for ICH are limited to recommendations on blood pressure reduction, intracranial pressure monitoring, osmotherapy with adequate fluid resuscitation, fever and glycemic control, and seizure prophylaxis [1]. Surgical approaches for managing ICH include traditional open surgical removal of the hematoma, endoscopic evacuation of the hematoma [2,3], key-hole craniotomy and minimally invasive stereotactic puncture and thrombolysis therapy [4]. Although the efficacy of surgical treatment of ICH remains unproven and controversial [5], and open surgery does not appear to improve the patient’s outcome, less invasive methods of hematoma evacuation seem to show promising results in improving patient outcome and survival [5]. Minimally invasive stereotactic puncture and thrombolysis therapy have emerged as a promising management strategy for ICH patients [4,6]. Recently published studies have demonstrated that minimally invasive procedures can successfully evacuate intracerebral hematomas and improve the outcome of patients [2,4,5,7-11]. The advantages of minimally invasive stereotactic puncture therapy are safety, and seemed to be feasible and to had a trend towards improved long-term outcome [4].

However, although the minimally invasive stereotactic puncture and thrombolysis therapy has displayed favorable outcomes, the effect of such procedure for ICH evacuation on perihematomal brain tissues remains elusive. The pathophysiological time window of performing such minimally invasive surgery is under investigation. Clinically, the early stage (within 7–24 h) was found to be the optimal time-window for surgical intervention of spontaneous ICH [12]. However, these results are based on clinical observations. The selected indices could not reflect the perihematomal pathophysiological changes.

The initial onset of ICH is always followed by a perihematomal brain edema formation which causes a secondary brain damage, leading to significant neurological deterioration [9]. The perihematomal brain edema formation after ICH is associated with blood–brain barrier (BBB) disruption induced by a range of factors, including matrix metalloproteinases and the excitatory neurotransmitter glutamate [10,13-15].

Matrix metalloproteinases (MMPs) have been demonstrated to be associated with BBB disruption after ICH [16-19]. The role of MMPs in hemorrhagic stroke appears critical for hematoma and brain edema growth as well as for neuronal death, which are understood as secondary brain injury and may have a considerable clinical impact [20]. Inhibition of MMP activity by using pharmacological anti-MMPs strategies may provide an approach to reduce ongoing edema after ICH [19]. However, attempts at MMP inhibition in spontaneous ICH have solely been made under experimental conditions and were associated with a wide range of possible side effects [20]. Reducing the release of MMP-9 by minimally invasive stereotactic puncture and thrombolysis for ICH evacuation might be another choice. The permeability of BBB decreased as MMP-9 expression decreased after intracerebral hematomas were evacuated by a minimally invasive procedure [10]. However, the effects of minimally invasive procedures performed at different stages of intracerebral hematoma evacuation on MMP-9 levels and their correlation with the perihematomal BBB permeability have been poorly investigated.

The purpose of the present study was to provide pathophysiological evidences for an optimal time window of the minimally invasive stereotactic puncture and thrombolysis ICH evacuation by investigating the effects of performing such procedures at different stages on MMP-9 changes and their correlation with the perihematomal permeability of BBB.

## Methods

### Materials

#### *Reagents*

The following reagents were used in this study: TRIZOL reagent, chloroform, isopropyl alcohol, 100% ethanol, 75% ethanol, RNA enzyme-free water, RNA enzyme-free glycogen, TE (10 mM, Tris–HCl pH 8, 1 mM EDTA) (Tris–HCl, EDTA), 0.4 M MOPS, pH 7.0 (MOPS), 0.1 M sodium acetate, 0.01 M EDTA, Brominated b pounds and AGAR sugar were provided by Sangong Biotech (Shanghai) Co., Ltd; SuperScript. III First-Strand Synthesis System (Invitrogen), Hot Start Fluorescent PCR Core Reagent Kits (BIO BASIC INC, SYBR Green I), Formamide (molecular formula: HCONH_2_, Chongqing Chuanjiang Chemical Reagent Factory), urethane (molecular formula: C_3_H_7_NO_2_, Wuxi Yangshan Biochemical), Evans blue (Beijing Hengye Zhongyuan Chemical), 4% paraformaldehyde (Wuhan Boster Biological Technology), urokinase (Guangdong Livzon Pharmaceutical) were used in this study.

#### *Instruments*

For this study, we used the following instruments: a ZH-Lanxing B-Type rabbit stereotaxic Apparatus (Huaibei Zhenghua Biological Instrument & Equipment), electronic scales (Satourious, Germany), a Rainbow Type-722 grating spectrophotometer (Shandong Gaomi Rainbow Analytical Instrument), a 5415R high-speed centrifuge (Frozen, Heraeus Company), micropipettors (Eppendorf), a 202–2 constant temperature oven (Shanghai Luda Laboratory Apparatus), a digital display thermostat water bath HH-2 (Guohua Electric Appliance), a desktop general centrifuge (TGL-16B; Shanghai Anting Scientific Instrument Factory), a -80°C freezer (Forman Scientific Company), a refrigerator (Qingdao), a CT provided by the Guiyang Medical College, a G1315 A diode-array detector (DAD, Agilent Technologies, USA), a pH meter (410 A, ORION, USA), an Agilent 1313A Automatic Sampler (Agilent Technologies, USA), a column oven (Agilent Technologies, USA), scales (Beijing Gangdong Hengye Instrument), Fluorescence quantitative PCR instrument (ABI 7500 Fast), Polymerase chain reaction (PCR) amplification instrument (BBI Canada), SW-CJ-1 D clean bench (Jiangsu Sue clean the equipment factory), DK-8 D type the electrical thermostatic sink (Shanghai Senxin Experiment Instrument Co., LTD), DYY-8 voltage type stablized electrophoresis apparatus (Shanghai Qi’s Analysis Instrument Co., LTD), YXJ-2 centrifuge (Hunan Instrument Centrifuge Instrument Co., LTD), H6-1 miniature electrophoresis slot (Shanghai Lean Organic Glass Instrument Plant); Gel imaging system (Gene Genius), U-3010 UV–vis spectrophotometer (Hitachi), Removing liquid device (range 100–1000 ml, 20–200 ml, 0.5–10 ml) (Canada BBI), and Primer design software: Primer Premier 5.0.

### Experimental groups

The present study was approved by the Animal Care and Use Committee of Guiyang Medical College.

Sixty male rabbits (2.8–3.4 kg) were provided by the Animal Center of Guiyang Medical College. These rabbits were randomly assigned to a model control group (MC group, including 30 rabbits), or a minimally invasive treatment group (MI group, including 30 rabbits). Both the MC group and the MI group were equally divided into 5 subgroups; each subgroup included 6 rabbits. An ICH was induced in all animals in the MC and the MI groups.

### Animal preparations

#### *ICH model preparation*

**Figure 1:**
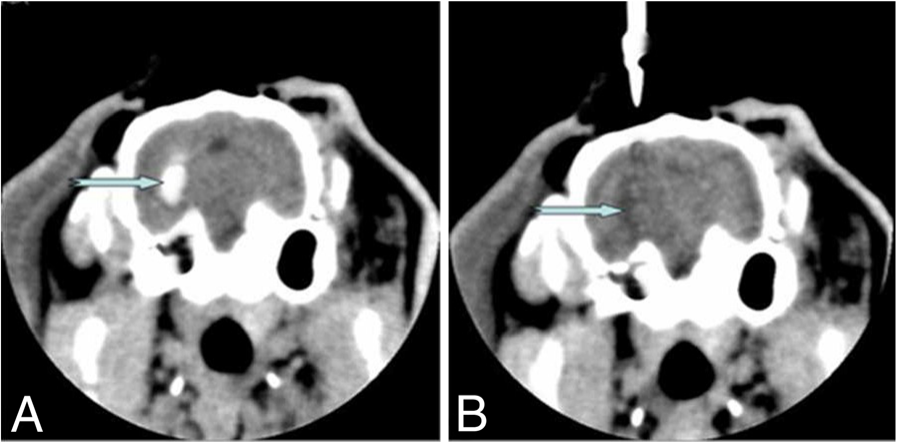
**Brain CT showing changes of intracranial hematoma before and after the minimally invasive procedures. A**: Basal ganglia hematoma was observed after the ICH model was prepared successfully. **B**: The intracerebral hematoma disappeared after the MI procedures were performed.

The method used to prepare an ICH model was the same as that used in our previously published studies [10,21,22]. Briefly, the rabbits were fastened to the stereotaxic apparatus. The head was then adjusted to make bregma 1.5 mm higher than the lambdoid suture. The skull of the rabbit was drilled, and using a #12 needle and a 1-ml syringe, 0.5 ml autologous arterial blood was taken from the central ear artery. The syringe was then connected to a #7 needle from which the tip was removed. The #7 needle was then inserted vertically and quickly into the skull 12 mm deep, and the blood was slowly injected into the basal ganglia. A CT scan was performed 3 hours later. A high-density shadow in the basal ganglia region with no shadow in the lateral ventricle was considered to be successful ICH induction (Figure 1A).

The rabbits were sent back to the animal room and housed as usual after successful ICH induction confirmed by CT scan. All of the animals recovered from anesthesia within 5 hours after intravenous injection of 20% urethane. The total anesthesia time was 3–5 hours.

The exclusion criteria included visualization of back flow along the needle track, blood in the ventricle, and death of the rabbit.

#### *Evaluation of the ICH volume*

At 3 days after the ICH model was established, a CT scan in all the rabbits was performed again to demonstrate the efficacy of the surgical procedures. The volume of ICH was calculated using the Tada formula [π /6 × length (cm) × width (cm) × height (cm)] before MI surgery. Histological sections were prepared in all animals to observe the hematoma volume when the rabbits were sacrificed two weeks after the minimally invasive surgery was performed.

#### *Minimally invasive procedures for ICH evacuation*

The rabbits from the MI and MC groups were anesthetized again by injecting 20% urethane (5 ml/kg) into the ear vein. They were then placed in the stereotaxic apparatus, a #7 needle was inserted into the hematoma.

In MI group the liquid part of the hematoma was aspirated. We then injected 5,000 U of urokinase (dissolved in 0.5 ml of 0.9% sodium chloride solution) into the hematoma. The needle was kept in place for 15 min, followed by slow aspiration while withdrawing the needle. The rabbits were then placed back in the breeding room for two weeks.

A CT scan was performed again three days after the surgical procedures to demonstrate the efficacy of minimally invasive procedures.

Procedures performed in the MI group were also performed in the MC group, but just with a sham evacuation without aspirating the hematoma, and without injecting the urokinase into the hematoma.

#### *Medical treatment of the animals*

Animals in each group only received an intramuscular injection of penicillin (400,000 U) to prevent infection, and they were housed as usual until they were sacrificed. No other medical treatment was administered.

### Neurological deficit score measurement

A neurological deficit scale was used to determine neurological function between groups [23]. The scale included tests of motor function (1–4), consciousness (1–4), head turning (0–1), circling (0–1) and hemianopsia (0–1). A total score of 11 indicates maximum impairment (comatose or dead rabbit), whereas 2 denotes complete normality. Tests were conducted by an observer blinded to the animals.

### Brain tissues preparation

Two weeks after the hematoma was evacuated by the MI procedures, the animals were sacrificed to obtain the brain tissues for detecting the MMP-9 and the EB content. The brain was then extracted and placed on ice. Using the needle track as the center to prepare a coronal section and a sagittal section, then the brain on the hematoma side was cut and divided into four parts: anterior-inner, anterior-outside, posterior-inner and posterior-outside. A total of 5 mm of brain tissue surrounding the hematoma was collected from each part mentioned above. The anterior-inner part was used for MMP-9 mRNA detection and was stored at -80°C. The anterior-outside part was used for testing the Evans blue content, while the posterior-inner part and posterior-outside parts were used for testing the water content in the brain.

### Real Time-PCR for MMP-9 mRNA detection

The animals were sacrificed, and the brains were removed. Brain samples (approximately 50 mg) extracted from the anterior-outside part of the hematoma were pulverized [24], and total RNA was isolated using TRIzol reagent. cDNAs were generated from 8 μl (0.5 μg/μl) of total RNA by Superscript II RNase H- reverse transcriptase primed with oligo (dT) 1 μl (0.5 μg/μl). All primers for MMP-9, β-actin and the PCR protocol were designed as follows: MMP9-F: 5′ACTTCCAACTTTGACAGCGAC3′ (Tm = 56.9), MMP9-R: 5′-GAGTGATCCAAGCCCAGTG3′ (Tm = 55.4 110 bp); β-actin primers: 5′-GGTCATCACCATCGGCAAC3′ (Tm = 58.7), β-actin: 5′-ATGTCCACGTCGCACTTCA 3′ (Tm = 57.4 129 bp). Multitarget PCRs were performed by coamplifying β-actin as an internal standard. The reaction component included Hotstart fluo-PCR mix 12.525 μl, primer F 0.5 μl, primer R 0.5 μl, ddH20 9.5 μl and cDNA 2 μl. The reaction was performed at 95°C for 2 min per 1 cycle. Each of 40 cycles included 95°C, 10 s; 58°C (β-actin) and 56°C (MMP-9), 30 s, and 68°C, 40 s.

A ∆Ct value was used to determine the relative quantity of MMP-9 mRNA. The ∆Ct value reflects the difference between the value of sample mRNA expression and the R-β-actin mRNA expression. The greater the ∆Ct value is, the less the sample mRNA.

### The BBB permeability measurement

Evans blue was used as a tracer to measure the BBB permeability. Two hours before each experiment, 2% Evans blue (2 ml/kg) was injected into the ear vein. After 2 h, the brain tissue was quickly removed. The tissue surrounding the hematoma was weighed (with an accuracy of 0.1 mg) and then placed into a test tube with 4 ml of formamide. The tube was then capped and placed in a 54°C water bath for 24 h to allow the Evans blue to spread throughout the brain tissue. The samples were then centrifuged at 2400 rpm for 5 min. A spectrophotometer was used (λ = 632 nm) to measure the absorbance of the supernatant, which was removed using a straw and placed into a quartz cuvette. Absorbency was measured using formamide alone as a blank control.

Evans blue (4 mg) was placed into a volumetric flask and weighed (within the accuracy of 0.1 mg). A total of 100 ml NS was added, and the solution was stirred. From this solution, 0.3 ml was removed and placed in 5.7 ml of formamide to make the standard buffer solution. A total of 3 ml of this solution was serially diluted in tubes, each containing 3 ml of formamide. The amount of Evans blue in each of the seven tubes was 8, 4, 2, 1, 0.5, 0.25 and 0.125 μg per ml. The tubes were capped and placed into a 54°C water bath for 24 hours. The aforementioned method to measure absorbance was then used. Linear regressions were then calculated for the absorbencies and Evans blue content. The final equation was y = 0.0053 x +0.0608 (R^2^ = 0.9833).

We used the formamide method to measure the Evans blue content in the brain tissue to gauge the severity of BBB damage. The formula used was as follows: Evans blue content in brain tissue (μg/g wet brain) = B × formamide (ml)/wet weight (g), where B refers to the Evans blue content of the sample (μg/ml) given by the linear regression equation according to standard curve.

### The brain water content measurement

The dry and wet weight method was used to measure the water content of the brain tissue. The brains were quickly removed, and a total of 5 mm of brain tissue surrounding the hematoma was collected. The brain tissue from the posterior-inner part and the posterior-outside parts of the hematoma were used for testing the WBC.. First, the weight of the wet tissue was obtained. The samples were placed in an oven at 100°C for 48 h, and the dried samples were then weighed. The water content of the brain tissue was calculated as (wet weight – dry weight)/wet weight × 100%.

### Statistical analysis

All data were analyzed using SPSS 11.5. Basic data are expressed as the mean ± standard deviation (X ± SD). A repeated measures ANOVA was used to make comparisons across the entire time series. When a difference was detected by ANOVA, a q test or Bonferroni was used to make comparisons between every two groups. A *p* value less than 0.05 was considered to be statistically significant. Statistical analysis was performed in consultation with the Department of Biostatistics of Guiyang Medical College.

## Results

### ICH model preparation

Following the blood was infused into the basal ganglia, the animals were unable to stand up or crawl. The contralateral extremities were less responsive to noxious stimulation. Brain CT showed an oval or round hyperdensity in the basal ganglia (Figure 1A), demonstrating that the ICH model in this study was successful and reliable. The ICH model was successfully produced in the MC group and the MI group. All animals were kept alive until the experiment was terminated. A total of 60 rabbits (30 rabbits in the MC group, and 30 ones in the MI group) were included in the present study.

### Changes of ICH volume after surgery

**Figure 2:**
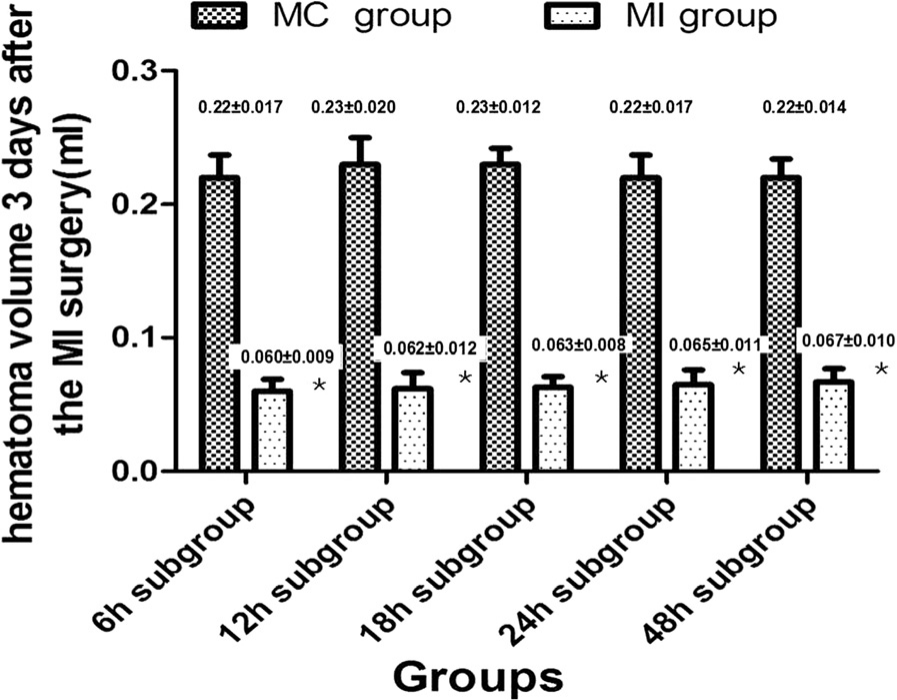
**The hematoma volume changes after the minimally invasive surgery.** The hematoma volume decreased significantly in different MI subgroups compared with the MC group, suggesting that the minimally invasive surgery could effectively evacuate the ICH.

A brain CT showed that there were no significant differences in the hematoma volume between the MI (0.478±0.025 ml) and the MC groups (0.480±0.028 ml) after the ICH model was prepared successfully. The volume of ICH remained unchanged in the MC group when the animals experienced a repeated CT scan. However, the brain CT showed that the hematoma was evacuated almost completely in all MI subgroups (Figure 1B). A significant difference in the ICH volume was observed between the MC group (0.406±0.032 ml) and the MI group (0.024±0.003 ml), suggesting that the MI procedures could successfully evacuate the intracerebral hematoma (Figure 2).

### The changes of the functional neuroscore

**Figure 3:**
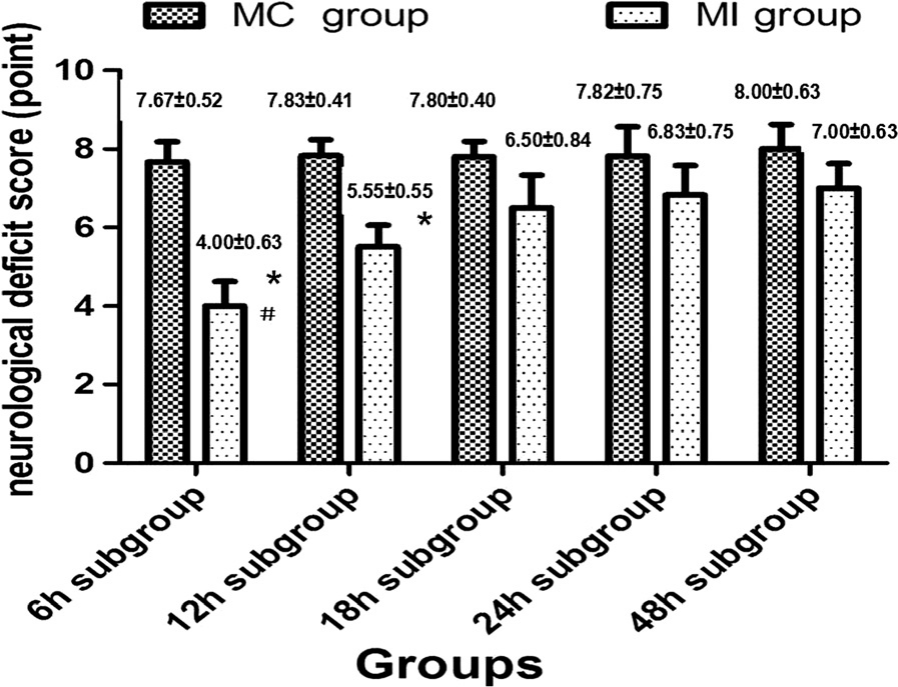
**The neurofunctional score after MI procedures in different time window.** The neurological deficit score decreased significantly in different MI subgroups compared with the MC group. A significant difference of the neurological deficit score in the MI group was also observed among subgroups. The 6 h group showed the most favorable outcome as compared with the other MI subgroups.

After the model of ICH was established successfully, the neurological deficit scores increased significantly (8.834±0.753) compared with the normal reference values (2.000±0.233), suggesting that the model produces a neurological deficit. In the MI group, however, the neurological deficit score decreased in each subgroup two weeks after the MI procedures were performed to evacuate the ICH (t=8.276, P<0.05) compared with the MC group. A significant difference was also observed among different MI subgroups (F=28.42); the 6–12 hour subgroup displayed a favorable outcome, suggesting that the MI surgery performed in early stages after ICH could effectively improve the neurofunctional outcome (Figure 3).

### Changes of perihematomal MMP-9 mRNA levels

**Figure 4:**
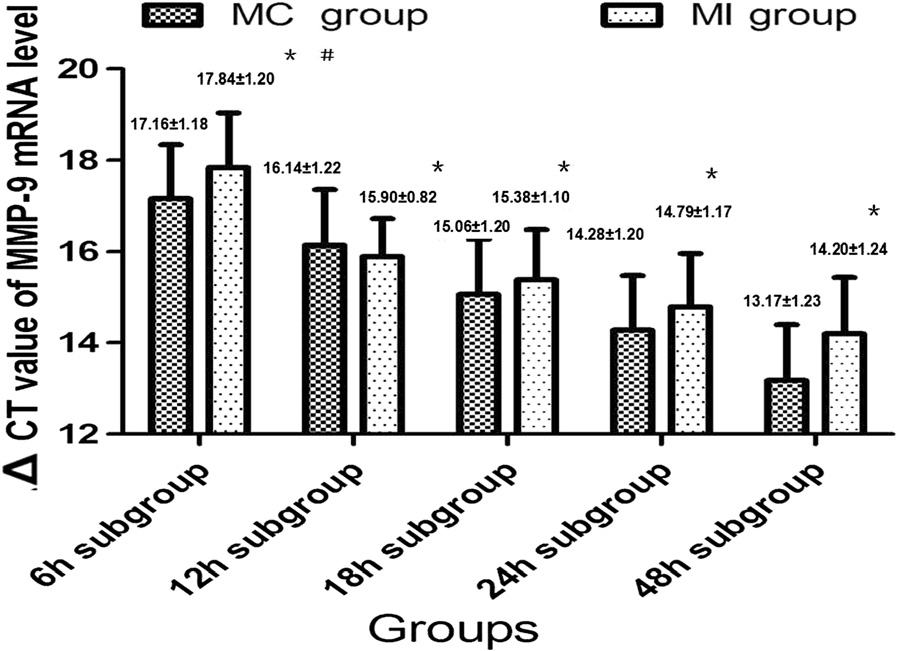
**∆Ct values changes of MMP-9 mRNA among different groups.** The ∆Ct values of perihematomal MMP-9 mRNA in different MI subgroups increased significantly as compared with the MC group. A significant difference of the MMP-9 mRNA was also observed among subgroups. These results suggested that the MI procedures for hematoma evacuation could decrease the quantity of MMP-9 mRNA. Performing the MI procedures in early stages after ICH displayed a favorable outcome.

Compared with the MC group, the quantity of the perihematomal MMP-9 decreased after the hematoma was evacuated by the MI procedures. However, the quantity of perihematomal MMP-9 mRNA was different among the MI subgroups (F=65.83, P<0.05). The quantity of perihematomal MMP-9 mRNA was lowest in the 6 h group, it increased gradually as the time window increased, and it reached the highest level in the 48 h group. A significant difference was also observed in the quantity of perihematomal MMP-9 mRNA between all subgroups in the MI group. The decrease of MMP-9 mRNA level in the 6 h and 12 h subgroups was more significant compared to that of the other subgroups (P<0.05), suggesting that the MI procedures for evacuating intracerebral hematoma in early stages could significantly reduce the perihematomal MMP-9 mRNA content (Figure 4).

### The changes of BBB permeability and brain water content

**Figure 5:**
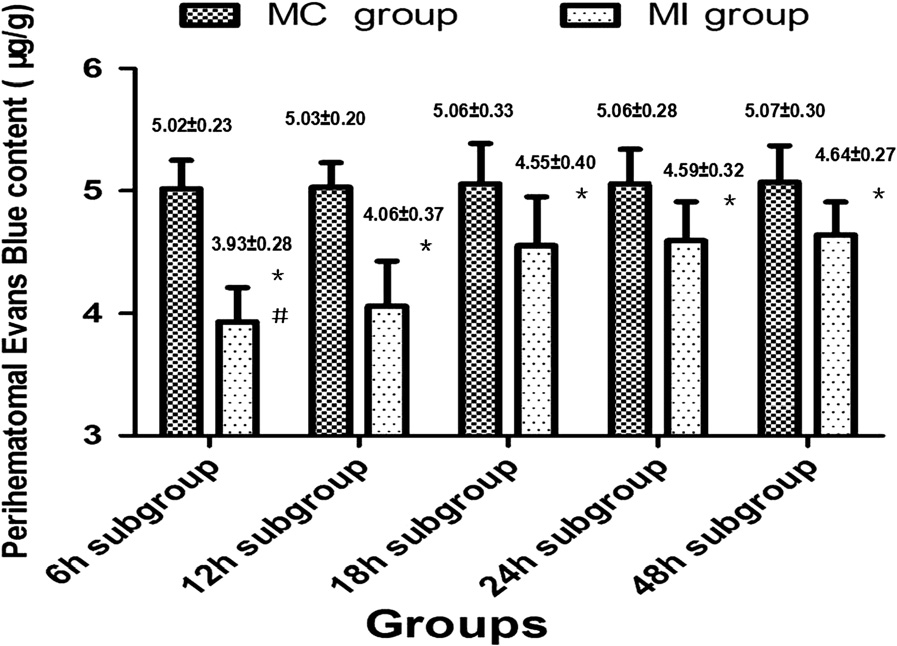
**Perihematomal Evans blue content changes before and after the MI procedures.** Perihematomal Evans blue content significantly decreased after the hematoma was evacuated by the MI procedures. The perihematomal Evans Blue content was varied in different time MI subgroups. Performing the MI procedures in 6–12 hours after ICH showed the most favorable results.

**Figure 6:**
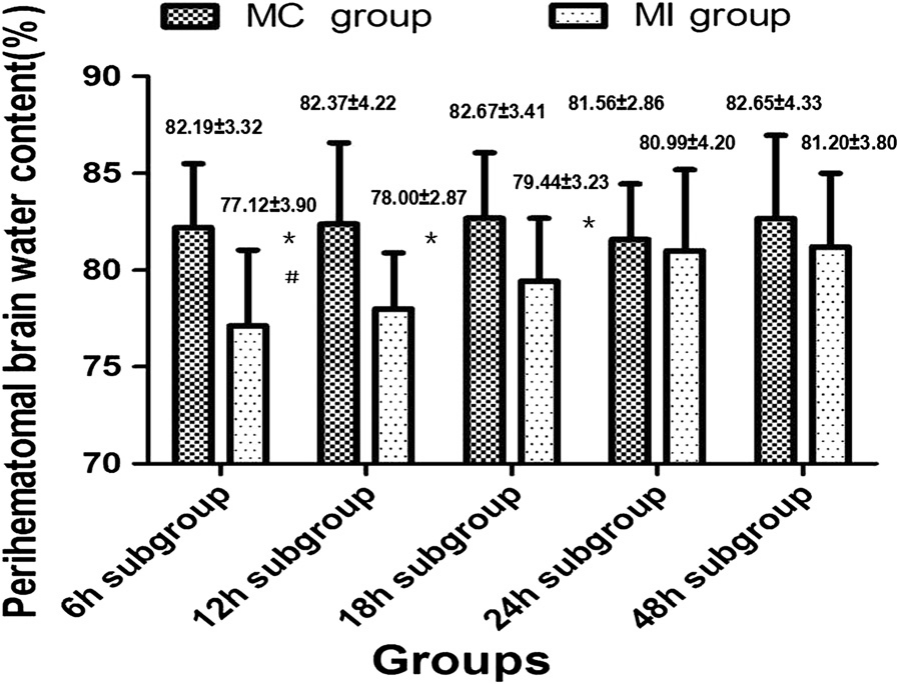
**Perihematomal BWC changes before and after the MI procedures.** Perihematomal BWC significantly decreased in different MI subgroups as compared with the MC group. The perihematomal BWC was varied in different time MI subgroups. Performing the MI procedures in 6–12 hours after ICH showed the most favorable results.

The perihematomal Evans blue content (F=93.85, P<0.05) and BWC (F=1089.50, P<0.05) were significantly changed across different times within the MI subgroups. Both the Evans blue and the BWC in each subgroup were decreased significantly as compared with the MC group. Among the MI subgroups, the perihematomal Evans blue content and the BWC were lowest in the 6 h MI group. As the time window prolonged, Evans blue and water content increased and reached their peak in the 48 h MI group. These results suggested that evacuating the intracerebral hematoma by performing MI procedures within 48 hours after the onset of hemorrhage could reduce the perihematomal permeability of BBB and decrease cerebral edema. However, the secondary brain injury became more serious over time (Figures 5 and 6).

### Correlation of BBB permeability and MMP-9 mRNA

Both BBB permeability and the quantity of MMP-9 mRNA increased in the MC group as the time went on. The quantity of MMP-9 mRNA of MI subgroups decreased significantly compared to the MC group at each time point. The BBB permeability also decreased corresponding to the decreased quantity of MMP-9. The quantity of MMP-9 mRNA was lowest in the 6 h group and highest in the 48 h group, the same changes were also observed in BBB permeability experiments. A positive correlation between BBB permeability and the quantity of MMP-9 mRNA was noted (r=0.982, P<0.05).

## Discussions

Intracerebral hemorrhage is a major public-health problem worldwide [25]. Larger ICHs are typically associated with high mortality and morbidity, leaving survivors with severe neurological disability. Many questions remain unanswered regarding the clinical management of ICH, although the medical and surgical clinical trials completed in the past 10 years have refined our understanding of the goals of ICH management [25]. Recently published studies have demonstrated that minimally invasive procedures can successfully evacuate intracerebral hematomas and improve the outcome of patients [2,4,5,7-11]. Minimally invasive stereotactic puncture and thrombolysis therapy have emerged as a promising management strategy for ICH patients [4,6]. However, the pathophysiological time window for minimally invasive surgery remains to be elucidated. We are unable to determine this time window in humans. Large animal models of ICH might provide an important tool for physicians to observe the effect of minimally invasive surgery on the perihematomal changes. Previously published studies have demonstrated that the perihematomal pathophysiological changes are associated with a range of substances such as inflammatory mediators, thrombin, hemoglobin breakdown products, oxidative stress, complement, and matrix metalloproteinases [19,20,26]. Matrix metalloproteinases (MMPs) have been implicated in BBB disruption and ICH pathogenesis [27-30]. Several studies have demonstrated that MMP-9 plays an important role in BBB disruption after stroke [27,28,31]. Therefore, administering MMP-9 antagonist to reduce its effect might be beneficial for protecting BBB from disruption [19,32]. However, MMP inhibition in spontaneous ICH has solely been made under experimental conditions, and the basic research has yet to yield significant advancements for clinical practice. So, reducing the production of perihematomal MMP-9 by surgical procedures for hematoma evacuation might be a better option. Standard open craniotomy for clearance of intracerebral hematoma often causes damage to the uninjured brain tissue overlying the hematoma. Minimally invasive surgery combines the benefits of surgical clot removal with limited tissue damage and shorter surgery duration [33]. In the present study, we prepared a rabbit model of ICH to observe the impact of performing the minimally invasive procedure at different stages on perihematomal MMP-9 and its correlation with BBB permeability. The results showed that perihematomal MMP-9 significantly decreased after surgery within different time windows for the evacuation of intracerebral hematoma as compared with the MC group. The quantity of perihematomal MMP-9 was different among the MI subgroups. It was lowest in the 6 h group, and increased gradually as the time window prolonged, reaching the highest level in the 48 h group. The decrease of MMP-9 level in the 6 h and 12 h subgroups was more significant compared to that of the other subgroups, suggesting that the MI procedures for evacuating intracerebral hematoma in early stages could significantly reduce the perihematomal MMP-9 content. The Evans blue content also deceased at each time point as compared with the MC subgroups, suggesting that the permeability of BBB were decreased corresponding to the decreased MMP-9.

Regarding the neurological function, the MI group displayed a significant decreased neurological deficit score two weeks after ICH model preparation compared with the MC group. The 6–12 hour MI subgroup displayed a favorable outcome [22,34,35], suggesting that the MI surgery performed in early stages after ICH could effectively improve the neurofunctional outcome.

Earlier therapeutic time window (0–6 h) would be helpful in reducing the perihematomal secondary brain damages and improving neurological functions. But in clinical practice, the 6–24 h window is usually the earliest surgical procedures can be performed. The interval between the onset of ICH and the ICH evacuation is prolonged by patient presentation to the hospital, diagnostic work-up, procedures for randomization, arrangements for stereotactic navigation, and so on. So we selected 6 h as one of the time points to observe the outcome of neurofunction and the perihematomal changes.

The effects of performing the minimally invasive surgery in super-early stage (0-6 h) on the outcome of treatment were not observed in the current study. This would be a limitation of the present study. A further experimental study would be required for observing the effect of performing the minimally invasive surgery in 3–6 h after ICH to evacuate the hematoma on the outcome of neurofunction, and the perihematomal pathophysiological changes.

## Conclusions

These data indicated that performing the MI procedures within 6–12 hours displayed a significant reduction of MMP-9 and Evans blue content. As the time window was prolonged, the effects of the MI procedures on MMP-9 decreased compared with the 6 h–12 h subgroup. The pathophysiological time window for the MI surgery might be within 6–12 hours after ICH without considering the issue of re-hemorrhage.

## Competing interests

The authors declare that they have no competing interests.

## Authors’ contributions

GW conceived of the study, and participated in its design and coordination and drafted the manuscript. JS prepared the model of ICH. FW and AF conducted the experimental study and the statistical analysis. LW participated in the design of the study and helped to draft the manuscript. SR helpted to performed the statistical analysis. All authors read and approved the final manuscript.

## Pre-publication history

The pre-publication history for this paper can be accessed here:

http://www.biomedcentral.com/1471-2377/14/85/prepub

## References

[B1] Rincon F Mayer SA Current treatment options for intracerebral hemorrhage Curr Treat Options Cardiovasc Med 2008 10 3 229 240 18582412 10.1007/s11936-008-0025-x

[B2] Miller CM Vespa P Saver JL Kidwell CS Carmichael ST Alger J Frazee J Starkman S Liebeskind D Nenov V Elashoff R Martin N Image-guided endoscopic evacuation of spontaneous intracerebral hemorrhage Surg Neurol 2008 69 5 441 446 discussion 446 18424298 10.1016/j.surneu.2007.12.016 PMC4160887

[B3] Orakcioglu B Uozumi Y Unterberg A Endoscopic intra-hematomal evacuation of intracerebral hematomas - a suitable technique for patients with coagulopathies Acta Neurochir 2011 112 3 8 10.1007/978-3-7091-0661-7_1 21691979

[B4] Zhou H Zhang Y Liu L Huang Y Tang Y Su J Hua W Han X Xue J Dong Q Minimally invasive stereotactic puncture and thrombolysis therapy improves long-term outcome after acute intracerebral hemorrhage J Neurol 2011 258 4 661 669 21340523 10.1007/s00415-011-5902-7 PMC3065646

[B5] Zuccarello M Andaluz N Wagner KR Minimally invasive therapy for intracerebral hematomas Neurosurg Clin N Am 2002 13 3 349 354 12486924 10.1016/s1042-3680(02)00008-6

[B6] Xu F Tang Z Luo X Kang H Hu Q Wang W Zhu S No evidence of preoperative hematoma growth representing an increased postoperative rebleeding risk for minimally invasive aspiration and thrombolysis of ICH Br J Neurosurg 2010 24 3 268 274 20465455 10.3109/02688691003624588

[B7] Barrett RJ Hussain R Coplin WM Berry S Keyl PM Hanley DF Johnson RR Carhuapoma JR Frameless stereotactic aspiration and thrombolysis of spontaneous intracerebral hemorrhage Neurocrit Care 2005 3 3 237 245 16377836 10.1385/NCC:3:3:237

[B8] Sun H Liu H Li D Liu L Yang J Wang W An effective treatment for cerebral hemorrhage: minimally invasive craniopuncture combined with urokinase infusion therapy Neurol Res 2010 32 4 371 377 20483003 10.1179/016164110X12670144526147

[B9] Miller CM Vespa PM McArthur DL Hirt D Etchepare M Frameless stereotactic aspiration and thrombolysis of deep intracerebral hemorrhage is associated with reduced levels of extracellular cerebral glutamate and unchanged lactate pyruvate ratios Neurocrit Care 2007 6 1 22 29 17356187 10.1385/NCC:6:1:22

[B10] Wu G Wang L Hong Z Li C Long X Sheng F Effects of minimally invasive procedures for removal of intracranial hematoma on matrix metalloproteinase expression and blood–brain barrier permeability in perihematomal brain tissues Neurol Res 2011 33 3 300 306 20712922 10.1179/016164110X12759951866993

[B11] Wu G Wang L Hong Z Mao Y Hu X Effects of minimally invasive techniques for evacuation of hematoma in basal ganglia on cortical spinal tract from patients with spontaneous hemorrhage: observed by diffusion tensor imaging Neurol Res 2010 32 10 1103 1109 20483024 10.1179/016164110X12656393665008

[B12] Wang YF Wu JS Mao Y Chen XC Zhou LF Zhang Y The optimal time-window for surgical treatment of spontaneous intracerebral hemorrhage: result of prospective randomized controlled trial of 500 cases Acta Neurochir 2008 105 141 145 10.1007/978-3-211-09469-3_29 19066100

[B13] Keep RF Xiang J Ennis SR Andjelkovic A Hua Y Xi G Hoff JT Blood–brain barrier function in intracerebral hemorrhage Acta Neurochir 2008 105 73 77 10.1007/978-3-211-09469-3_15 19066086

[B14] Wu G Wang L Liu J Mao Y Qin G Minimally Invasive Procedures Reduced the Damages to Motor Function in Patients with Thalamic Hematoma: Observed by Motor Evoked Potential and Diffusion Tensor Imaging J Stroke Cerebrovasc Dis 2013 22 3 232 240 21917481 10.1016/j.jstrokecerebrovasdis.2011.08.005

[B15] Hazell AS Excitotoxic mechanisms in stroke: an update of concepts and treatment strategies Neurochem Int 2007 50 7–8 941 953 17576023 10.1016/j.neuint.2007.04.026

[B16] Kawakita K Kawai N Kuroda Y Yasashita S Nagao S Expression of matrix metalloproteinase-9 in thrombin-induced brain edema formation in rats J Stroke Cerebrovasc Dis 2006 15 3 88 95 17904058 10.1016/j.jstrokecerebrovasdis.2006.01.002

[B17] Wu CH Huang FY Wang KY Huang SY Yang RL Li HZ Lei HX Lin JS Wang JM Yan XH [Expression of matrix metalloproteinase MMP-9 in the plasma and hematoma fluid of intracerebral hemorrhage patients] Zhonghua Yi Xue Za Zhi 2008 88 3 174 176 18361815

[B18] Xue M Fan Y Liu S Zygun DA Demchuk A Yong VW Contributions of multiple proteases to neurotoxicity in a mouse model of intracerebral haemorrhage Brain 2009 132 Pt 1 26 36 18772219 10.1093/brain/awn215

[B19] Xue M Yong VW Matrix metalloproteinases in intracerebral hemorrhage Neurol Res 2008 30 8 775 782 18826803 10.1179/174313208X341102

[B20] Florczak-Rzepka M Grond-Ginsbach C Montaner J Steiner T Matrix metalloproteinases in human spontaneous intracerebral hemorrhage: an update Cerebrovasc Dis 2012 34 4 249 262 23052179 10.1159/000341686

[B21] Wu G Li C Wang L Mao Y Hong Z Minimally invasive procedures for evacuation of intracerebral hemorrhage reduces perihematomal glutamate content, blood–brain barrier permeability and brain edema in rabbits Neurocrit Care 2011 14 1 118 126 21161434 10.1007/s12028-010-9473-8

[B22] Wu G Sheng F Wang L Wang F The pathophysiological time window study of performing minimally invasive procedures for the intracerebral hematoma evacuation in rabbit Brain Res 2012 1465 57 65 22658751 10.1016/j.brainres.2012.04.005

[B23] Purdy PD Devous MD Sr Batjer HH White CL 3rd Meyer Y Samson DS Microfibrillar collagen model of canine cerebral infarction Stroke 1989 20 10 1361 1367 2799867 10.1161/01.str.20.10.1361

[B24] Xi G Keep RF Hua Y Xiang J Hoff JT Attenuation of thrombin-induced brain edema by cerebral thrombin preconditioning Stroke 1999 30 6 1247 1255 10356108 10.1161/01.str.30.6.1247

[B25] Adeoye O Broderick JP Advances in the management of intracerebral hemorrhage Nat Rev 2010 6 11 593 601 10.1038/nrneurol.2010.146 20877400

[B26] Xue M Hollenberg MD Demchuk A Yong VW Relative importance of proteinase-activated receptor-1 versus matrix metalloproteinases in intracerebral hemorrhage-mediated neurotoxicity in mice Stroke 2009 40 6 2199 2204 19359644 10.1161/STROKEAHA.108.540393

[B27] Hernandez-Guillamon M Martinez-Saez E Delgado P Domingues-Montanari S Boada C Penalba A Boada M Pagola J Maisterra O Rodriguez-Luna D Molina CA Rovira A Alvarez-Sabin J Ortega-Aznar A Montaner J MMP-2/MMP-9 plasma level and brain expression in cerebral amyloid angiopathy-associated hemorrhagic stroke Brain Pathol 2012 22 2 133 141 21707819 10.1111/j.1750-3639.2011.00512.x PMC8029059

[B28] Koh SH Park CY Kim MK Lee KY Kim J Chang DI Kim HT Kim SH Microbleeds and free active MMP-9 are independent risk factors for neurological deterioration in acute lacunar stroke Eur J Neurol 2011 18 1 158 164 20550564 10.1111/j.1468-1331.2010.03100.x

[B29] Wang G Guo Q Hossain M Fazio V Zeynalov E Janigro D Mayberg MR Namura S Bone marrow-derived cells are the major source of MMP-9 contributing to blood–brain barrier dysfunction and infarct formation after ischemic stroke in mice Brain Res 2009 1294 183 192 19646426 10.1016/j.brainres.2009.07.070 PMC2758551

[B30] Rosell A Alvarez-Sabin J Arenillas JF Rovira A Delgado P Fernandez-Cadenas I Penalba A Molina CA Montaner J A matrix metalloproteinase protein array reveals a strong relation between MMP-9 and MMP-13 with diffusion-weighted image lesion increase in human stroke Stroke 2005 36 7 1415 1420 15947272 10.1161/01.STR.0000170641.01047.cc

[B31] Rosell A Cuadrado E Ortega-Aznar A Hernandez-Guillamon M Lo EH Montaner J MMP-9-positive neutrophil infiltration is associated to blood–brain barrier breakdown and basal lamina type IV collagen degradation during hemorrhagic transformation after human ischemic stroke Stroke 2008 39 4 1121 1126 18323498 10.1161/STROKEAHA.107.500868

[B32] Zhou J Li J Rosenbaum DM Barone FC Thrombopoietin protects the brain and improves sensorimotor functions: reduction of stroke-induced MMP-9 upregulation and blood–brain barrier injury J Cereb Blood Flow Metab 2011 31 3 924 933 20877384 10.1038/jcbfm.2010.171 PMC3063625

[B33] Thiex R Future perspectives on the fibrinolytic therapy of intracerebral hemorrhages Cent Nerv Syst Agents Med Chem 2011 11 2 150 156 21521167 10.2174/187152411796011367

[B34] Wu G Li S Wang L Mao Y The perihematomal glutamate level is associated with the outcome of patients with basal ganglia hematomas treated by minimally invasive procedures Neurol Res 2013 35 8 829 836 23676149 10.1179/1743132813Y.0000000220

[B35] Wu G Zhong W Effect of minimally invasive surgery for cerebral hematoma evacuation in different stages on motor evoked potential and thrombin in dog model of intracranial hemorrhage Neurol Res 2010 32 2 127 133 19726015 10.1179/016164109X12478302362617

